# Water temperature-dependent degradation of environmental DNA and its relation to bacterial abundance

**DOI:** 10.1371/journal.pone.0176608

**Published:** 2017-04-27

**Authors:** Satsuki Tsuji, Masayuki Ushio, Sho Sakurai, Toshifumi Minamoto, Hiroki Yamanaka

**Affiliations:** 1 Graduate School of Science and Technology, Ryukoku University, 1–5 Yokotani, Seta Oe-cho, Otsu, Japan; 2 Joint Research Center for Science and Technology, Ryukoku University, Otsu, Japan; 3 Center for Ecological Research, Kyoto University, Hirano, Otsu, Japan; 4 PRESTO, Japan Science and Technology Agency, Kawaguchi, Japan; 5 Graduate School of Human Development and Environment, Kobe University, Tsurukabuto, Nada-ku, Kobe, Japan; 6 Faculty of Science and Technology, Ryukoku University, Yokotani, Seta Oe-cho, Otsu, Japan; University of Hyogo, JAPAN

## Abstract

Environmental DNA (eDNA) is DNA shed by organisms into surrounding environments such as soil and water. The new methods using eDNA as a marker for species detection are being rapidly developed. Here we explore basic knowledge regarding the dependence of the eDNA degradation rate on time and water temperature, and the relationship between eDNA degradation and bacterial abundance. This subject has not been well clarified, even though it is essential for improving the reliability of eDNA analysis. To determine the time- and water temperature-dependent degradation of eDNA, river water was sampled and eDNA concentrations were determined for ayu sweetfish (*Plecoglossus altivelis altivelis*) and common carp (*Cyprinus carpio*) at seven time points, over a 48-h period, and at three different water temperatures. The degradation of eDNA was modeled for each species using an existing exponential decay model with an extension to include water temperature effects. The degradation models were constructed for ayu sweetfish as *N*_*t*_ = 229,901.2 × *exp* [− (0.01062 × *k* − 0.07081) × *t*] and for common carp as *N*_*t*_ = 2,558.0 × *exp* [− (0.01075 × *k* − 0.07372) × *t*]. *N*_*t*_ is the DNA concentration at time *t* (elapsed time in hours) and *k* is the water temperature (°C). We also measured the concentration of eDNA derived from purified genomic DNA of the common carp, which was spiked into aquarium water without the target species, and we measured the bacterial abundance in the sample water after 12 and 24 h of incubation. Environmental DNA degradation was accelerated at higher water temperatures (generalized linear model, GLM; *p* < 0.001), but bacterial abundance did not have a significant effect on eDNA degradation (GLM, *p* = 0.097). These results suggest that the proper treatment of this temperature effect in data interpretations and adjustments would increase the reliability of eDNA analysis in future studies.

## Introduction

Environmental DNA (eDNA) analysis is rapidly developing as a new tool for the biomonitoring of macroorganisms [1–4]. Environmental DNA is composed of DNA fragments shed by organisms into the surrounding water or soil. They are derived from various sources such as metabolic waste, damaged tissue, and sloughed skin cells [5,6]. Instead of capturing or observing the target organisms, scooping up a tank of water from a target site enables detection of the species inhabiting the site by using a specific region of the eDNA as a genetic marker. Ease of fieldwork is an advantage of eDNA analysis and this non-invasive method is nearly free from research-related impacts on the target species and their habitats. After the first application of eDNA analysis for species detection, Ficetola et al. [1], in which the American bullfrog (*Lithobates catesbeianus*) was detected in French wetlands, this method was expanded for use with various taxa and in various habitats [7–10]. Environmental DNA analysis has been applied not only for detection of species, but also for biomass estimations, as eDNA concentrations are positively correlated with biomass or abundance [9,11,12]. The shorter time requirements and cheaper costs of eDNA analysis enable researcheres to conduct long-term and large-scale observations more easily. Sigsgaard et al. [13] showed that the cost of the eDNA survey for detecting the nearly-extinct European weather loach (*Misgurnus fossilis*) in Denmark was almost half of the prospective cost for a conventional survey when they included the investigators’ salaries. Moreover, the time required for the eDNA survey was explicitly shorter than that required for a conventional survey. In addition, they achieved their goals and finally caught the loach with increased efforts, motivated by the detection of eDNA for this species at a specific location from a prior survey. Its outstanding detection capability, in addition to the easy on-site sampling and cost-effectiveness, makes eDNA analysis a prospective tool for natural resource management and ecological studies of biological communities [9,14].

Environmental DNA analysis is expected to become a powerful tool for biomonitoring. However, there are still some uncertainties about eDNA dynamics that could potentially result in significant errors regarding eDNA quantification. For example, it has been reported that eDNA is degraded rapidly over time after being released into the water from organisms [15,16] and that degradation rates were reported to have considerably large variation among studies [17]. Variation in eDNA degradation rates can be caused by variation in local environmental conditions, such as water temperature, pH, and light intensity [18–20]. Previous studies indicate that detailed knowledge of eDNA degradation is essential for improving estimations of initial eDNA concentrations at the sampling point and, therrby, improbing estimations of biomass.

Thomsen et al. [15] developed an eDNA degradation model based on time-dependent changes in the eDNA concentrations in sea water of the European flounder (*Platichthys flesus*) and the three-spined stickleback (*Gasterosteus aculeatus*). Many studies adopted the time-dependent exponential decay of eDNA as the model of degradation [16,17,19,20]; however, by only observing the time-dependent degradation of eDNA, their constructed models only incorporated time as a variable, without determining the effects of other environmental factors on degradation rates. It has been suggested that eDNA degradation is decelerated at low water temperatures due to lower bacterial activities [17,19,20]. This observation suggests potential effects of bacterial abundance on the degradation rate of eDNA and its dependence on temperature.

In most previous studies, tank experiments using well water [18], store-bought spring water [19], or tap water [16] were performed to determine the degradation rates of eDNA derived from individual target fish that were put into the aquariums. Although these experiments using artificial water provided helpful insight into the mechanisms of the eDNA degradation process, the properties of the water (dissolved organic compounds, suspended solids, and microbial abundance) were different from those of natural aquatic ecosystems. To the best of our knowledge, there have been only two reports that estimated eDNA degradation rates using field water [15,17]. Although the degradation rates could vary among habitats depending on water quality, microbial abundance, and water temperature, information on rates is currently available on from sea water and lake water samples [15,17]; additional information on degradation rates in different habitats would be desirable. Moreover, clarification on the relationship among water temperature, bacterial abundance, and degradation rates of eDNA would promote a better understanding of the effects of water quality on eDNA degradation.

The purpose of this study was to determine the water temperature-dependent degradation rate of eDNA shed by ayu sweetfish (*Plecoglossus altivelis altivelis* Temminck and Schlegel 1846; Plecoglossidae, Osmeriformes) and common carp (*Cyprinus carpio* Linnaeus 1758; Cyprinidae, Cypriniformes) by using water samples from a river inhabited by both species and to construct a refined nonlinear model that additionally incorporates the effect of water temperature in the existing degradation model. This would be a more versatile model that could estimate the initial concentration of eDNA at the sampling time when we need to correct for the degradation of eDNA during transportation of the water samples. We performed laboratory experiments using purified common carp DNA to determine the effects of bacterial abundance, water temperature, and the interactions of these two factors on the degradation of eDNA. Understanding these two related factors of eDNA degradation would help to elucidate eDNA dynamics under field conditions.

## Materials and methods

### Ethical statement

There was no need to obtain permission for conducting this research, including field water sampling and lab experiments. Taking a small volume of water from a public river is not prohibited. In lab experiments, a purified DNA sample from fish was used (Experiment 2); however, no animals were killed for use in this study because the DNA sample was an archived sample stocked in our lab from a previous study. The goldfish and dark chub used in Experiment 2 were maintained for use in an undergraduate practical training course for students who major in museology, not for this research. Moreover, the current laws and guidelines of Japan relating to animal experiments allow the usage of fish DNA samples without any ethical approvals from any authorities. All experiments in this study followed the current laws of Japan.

### Experimental design

In the present study, we performed two experiments. In Experiment 1, time- and water temperature-dependent degradation of eDNA in field water was monitored using concentration measurements of eDNA in water samples incubated at three different temperatures. In Experiment 2, the relationship between bacterial abundance and the eDNA degradation rate was examined using aquarium water as a source of bacteria and purified DNA of common carp as the eDNA source at three temperatures. To keep maintain relatively constant initial eDNA concentration across experimental replications, a known amount of purified common carp DNA was added to each water sample that was collected from an aquarium without common carp. The field water was not used because the water of Yasu River originally contained the eDNA of the common carp and would have produced a large unintended variation in eDNA concentrations among replications due to the heterogeneous distribution of eDNA in the field water. In this experiment, bacterial abundance would have been better controlled independently of temperature or time to determine its effect on eDNA degradation directly, i.e., spiking different amounts of bacteria into water samples to vary bacterial abundance in controls. However, we simply used the initial concentration of bacteria in the aquarium water for the incubation experiment due to difficulties in separating and concentration total bacteria from water. The methods described in the next two sections (Water filtration and DNA collection; Real-time quantitative PCR) were the same for both experiments. The temperature of the thermostatic water bath was kept constant during each experiment.

### Water filtration and DNA collection

All water samples were filtered using a Whatman GF/F filter (GE Healthcare Life Sciences, Piscataway, NJ, USA), with a diameter of 47 mm and a nominal pore size of 0.7 μm. Each filter disk was folded inward in half with tweezers and wrapped in aluminum foil, then placed in a plastic bag with a zipper and stored at –20°C until DNA extractions were performed. To prevent contamination between samples, all filtration instruments were cleaned before use by immersing in 10% bleach solution for 5 min, washing with running tap water, and then rinsing with Milli-Q water. Further, to monitor contamination from experimental equipment, Milli-Q water was filtered at the same volume as sample water at each sampling time point during the incubation experiment. Identical experimental steps were applied to both experimental samples and the Milli-Q water treatment.

DNA was extracted and purified using the protocol described by Tsuji et al. [6]. Each half-folded frozen filter was rolled into a cylindrical shape without unfolding and placed in the upper part of the spin column with 2.0-mL collection tubes (EZ-10, BioBasic, Ontario, Canada). Silica gel membranes, equipped to the EZ-10 spin column, were removed and discarded prior to use. Reagents from the DNeasy Blood and Tissue Kit (Qiagen, Manufacturer Location) were used with the EZ-10 spin columns (Bio Basic) for DNA extraction and purification. The spin columns were then centrifuged for 1 min at 6,000 × *g* to remove any excess water contained in the filter. Four hundred microliters of Milli-Q water, 200 μL of buffer AL, and 20 μL of proteinase K were mixed and dispensed onto the filter in each spin column, and the spin columns were incubated for 15 min at 56°C. After incubation, the spin columns were centrifuged for 1 min at 6,000 × *g*, and the eluted filtrate was transferred to a new 1.5-mL microtube. Four hundred microliters of Tris-EDTA buffer (pH 8.0) were added to each filter, and the filter was incubated for 1 min at room temperature before being centrifuged for 1 min at 6,000 × *g*. The upper part of the spin column containing the filter was removed from the 2.0-mL collection tube and the first and second filtrates were combined in the 2.0-mL tube. Then, 200 μL of buffer AL and 610 μL of ethanol were added to the combined filtrates and mixed well by gently pipetting up and down. The DNA in each mixture was purified with the DNeasy Blood & Tissue Kit (Qiagen) following the manufacturer’s instruction. Each mixture was transferred to a spin column provided by the DNeasy Blood & Tissue Kit to trap DNA fragments on its silica-gel membrane by centrifugation. Because of the large volume of each mixture, this step was repeated three times to catch all the DNA on the membrane. The silica-gel membrane was then washed twice with the washing buffers AW1 and AW2, and DNA was eluted from the column with 100 μL of buffer AE. The DNA extractions from filters were carried out within 24 and 48 hours after the filtration step in Experiments 1 and 2, respectively.

### Quantitative real-time PCR

Environmental DNA was quantified according to the method described by Takahara et al. [11]. Quantitative real-time TaqMan® polymerase chain reaction (PCR) was performed using a StepOne-Plus™ Real-Time PCR system (Applied Biosystems, FosterCity, CA, USA) to estimate copy numbers of the target DNA in each sample. A specific region of the eDNA from each target species was amplified by using the previously reported primers and probe sets: Paa-CyB-Forward (5′-CCTAGTCTCCCTGGCTTTATTCTCT-3′), Paa-CyB-Reverse (5′-GTAGAATGGCGTAGGCGAAAA-3′), and Paa-CyB-Probe (5′-FAM-ACTTCACGGCAGCCAACCCCC-TAMRA-3′) for the ayu sweetfish mitochondrial cytochrome *b* gene [10]; and CpCyB_496F (5′-GGTGGGTTCTCAGTAGACAATGC-3′), CpCyB_573R (5′-GGCGGCAATAACAAATGGTAGT-3′), and CpCyB_550p probe (5′-FAM-CACTAACACGATTCTTCGCATTCCACTTCC-TAMRA-3′) for the common carp mitochondrial cytochrome *b* gene [11]. It was confirmed that the primer and probe sets specifically detected DNA of the target species in the surveyed area [10,11].

Real-time PCR was performed in triplicate for each eDNA sample, standard dilution series, and PCR negative controls. PCR was conducted in 20-μL volumes and the reagent consisted of 900 nM of each primer, 125 nM of TaqMan® probe, plus sample DNA (the amounts of sample DNA used in each experiment are described below) in 1 × PCR master mix (TaqMan® Gene Expression Master Mix; Life Technologies, Carlsbad, CA, USA). The artificially-synthesized target sequence from ayu sweetfish DNA (399 bp) was cloned into qTAKN-2 plasmids, while the target sequence from common carp DNA was cloned into the pGEM plasmid. Both were used as standards for real-time PCR analyses for each species [10,11]. A standard dilution series containing 3 × 10^1^ to 3 × 10^4^ copies of the target sequences were analyzed in triplicate for each PCR test. For negative controls, instead of adding DNA template, the same volume of Milli-Q water was added to the PCR reactions. The PCR thermal conditions were as follows: 2 min at 50°C, 10 min at 95°C, then 55 cycles of 15 s at 95°C, and 60 s at 60°C. The R^2^ values of the standard curve ranged from 0.986 to 0.993 for ayu sweetfish and from 0.987 to 0.994 for common carp in Experiment 1 and from 0.984 to 0.988 for common carp in Experiment 2.

### Experiment 1: Water temperature-dependent degradation of eDNA

On July 22, 2014, 52 L of water was sampled from the surface of the Yasu River (35°2′23″N, 136° 1′19″E, Ritto, Japan) where ayu sweetfish and common carp were living. The water quality parameters measured were pH 7.54, temperature 26.1°C, and electrical conductivity 0.33 mS/cm, as determined by water quality sensors (HI 98128 pHep 5, HI 98312 DiST 6, and HI 98312 DiST 6, respectively; HANNA Instruments, Woonsocket, RI, USA). The sample waters were immediately transported on ice to the laboratory (amount of time required was 1 h 40 min). In the laboratory, 2 L of sample water was divided into four portions of 0.5 L each and filtered with a GF/F filter as the initial samples, and 0.5 L of Milli-Q water was filtered as an equipment blank. At the same time, the remaining sample water was dispensed into 12 tanks with 4 L of water each, and 4 tanks were assigned to each of three different incubation temperatures (mean ± standard deviation 10 ± 0.6°C, 20 ± 0.1°C, and 30 ± 0.2°C) maintained by a thermostatic water bath. The water temperature of each bath was recorded with a temperature logger during the experiment. After 1, 3, 6, 12, 24, and 48 h from the initiation of incubation, 0.5 L of water from each tank was sampled and filtered with a GF/F filter in the same manner as the initial sample. DNA extractions from the filters and quantification by real-time PCR were performed according to the method described above. The following amounts of extracted DNA were used as DNA template for each reaction in the real-time PCR: 2 μL for the ayu sweetfish experiment and 5 μL for the common carp experiment.

### Experiment 2: Relationship between eDNA degradation and bacterial abundance

On January 07, 2015, 3 L of surface water were sampled from an aquarium containing two non-target species; goldfish (*Carassius auratus* Linnaeus 1758; Cyprinidae, Cypriniformes) and dark chub (*Nipponocypris temminckii* Temminck and Schlegel 1846; Cyprinidae, Cypriniformes). The water quality parameters measured were pH 7.72, temperature 18.6°C, and electrical conductivity 0.15 mS/cm. The sample water was pre-filtered to retain particle sizes down to 6 μm and to remove large impurities such as residues of food and feces. The filtered water was expected to contain bacteria and was used as medium water in the following experiment. Purified total DNA from common carp, extracted from skeletal muscle tissue, was then spiked into the sample water and the mixture was stirred; the final DNA concentration was 0.5 ng/mL. In a subsequent incubation experiment, the concentration of common carp DNA and the bacterial abundance in the sample water were measured, along with the elapsed time. As the initial sample (*N*_*0*_) at time 0, 100 mL × 4 replications of the sample water were filtered with a GF/F filter. The remaining sample water was divided into 12 bottles with 215 mL in each, and 4 bottles were submerged in each of three thermostatic baths set at 10°C, 20°C, and 30°C. The water temperature in each thermostatic bath was recorded by temperature logger during the experiment. After 12 and 24 h, 100 mL of water from each of the four tanks in each of the three thermostatic baths were filtered with a GF/F filter. As an equipment blank, 100 mL of Milli-Q water was filtered at each time point. DNA extractions from the filters and quantification by real-time PCR were conducted according to the method described above. The following amounts of extracted DNA were used as DNA template for each reaction in the real-time PCR: 1 μL for the initial sample and 9 μL for the 12- and 24-h samples.

In parallel with the filtration, to investigate bacterial abundance in the sample water, microorganisms were cultured using the Standard Method Agar “Nissui” (code: 05618; Nissui Pharmaceutical, Tokyo, Japan) according to the manufacturer’s instructions. The composition of the Standard Method Agar per 1 L included: yeast extracts 2.5 g, peptone 5.0 g, glucose 1.0 g, and agar 15 g. The sample water was diluted fivefold with sterilized water. One milliliter of the diluted water sample was added to each of two petri dishes and then approximately 20 mL of the Standard Method Agar was admixed to coagulate. The Congealed Standard Method Agar was incubated for 24 h at 35°C. Afterward, the number of colonies was counted, and the average number of colonies between two replicated petri dishes were calculated as colony forming unit (CFU)/mL for each of six experimental conditions (two time periods [12 and 24 h] × three temperatures [10°C, 20°C, and 30°C]).

### Statistical analysis

All data on DNA concentration and microbial abundance were calculated as number of DNA copies per volume of filtered sample water and were used for statistical analysis. All statistical analyses were performed with R ver. 3.1.0 software [21].

In Experiment 1, the influence of time and water temperature on eDNA concentration was evaluated using generalized linear mixed-effects models (GLMM, package nlme) with a random effect of individual tanks. The resulting full model formula (shown in the conventional expression in R language) was lm (log [eDNA copies] ~ elapsed time + elapsed time: water temperature). Water temperature was incorporated into the model as an interaction term with time because the initial eDNA copies were the same for each water temperature. The coefficient of the interaction effect of time and water temperature at 10°C was set as 0. We compared the confidence intervals of the coefficient among the three temperature conditions using the glht function implemented in the “multcomp” package of R [22]. The minimum level of significance was set at *p* = 0.05. Then, referring to Thomsen et al. [15], the time-dependent eDNA decay model was extended to cover the water temperature-dependent effect.

The model is as follows:

$$\frac{dN}{dt}=-(a+bk)N$$ (1)

Solving this gives:

$$N_{t}=N_{0}\;\exp\left[-(bk+a)t\right]$$ (2)

The parameters are as follows: *N*_*t*_ is the DNA concentration at time *t* (hours). *N*_*0*_ is the initial DNA concentration, *k* is the water temperature (°C), *t* is the time (elapsed time in hours), and *a* and *b* are estimated by the nls function in R.

The half-decay time *t* (hour) was calculated by the following equation:

$$\frac{N_{0}}{N_{t}}=\frac{N_{0}}{N_{0}\exp[-(bk+a)t]}=2$$ (3)

Solving this gives:

t=ln(2)−(bk+a) (4)

In Experiment 2, the generalized linear model (GLM) of glm function in R was used to evaluate the influence of elapsed time, water temperature, and bacterial abundance on eDNA concentration. GLM was performed on the assumption of normal distribution, the log of common carp DNA concentration was set as the response variable, while the explanatory variables were elapsed time, water temperature, and bacterial abundance. Thus, the full model formula was glm (log [eDNA copies] ~ elapsed time + water temperature + bacterial abundance). GLM analysis was repeated to assess the effects of elapsed time and water temperature on bacterial abundance by setting bacterial abundance as the response variable and elapsed time and water temperature as explanatory variables. The full model formula was glm (bacterial abundance ~ elapsed time + water temperature).

## Results

**Fig 1:**
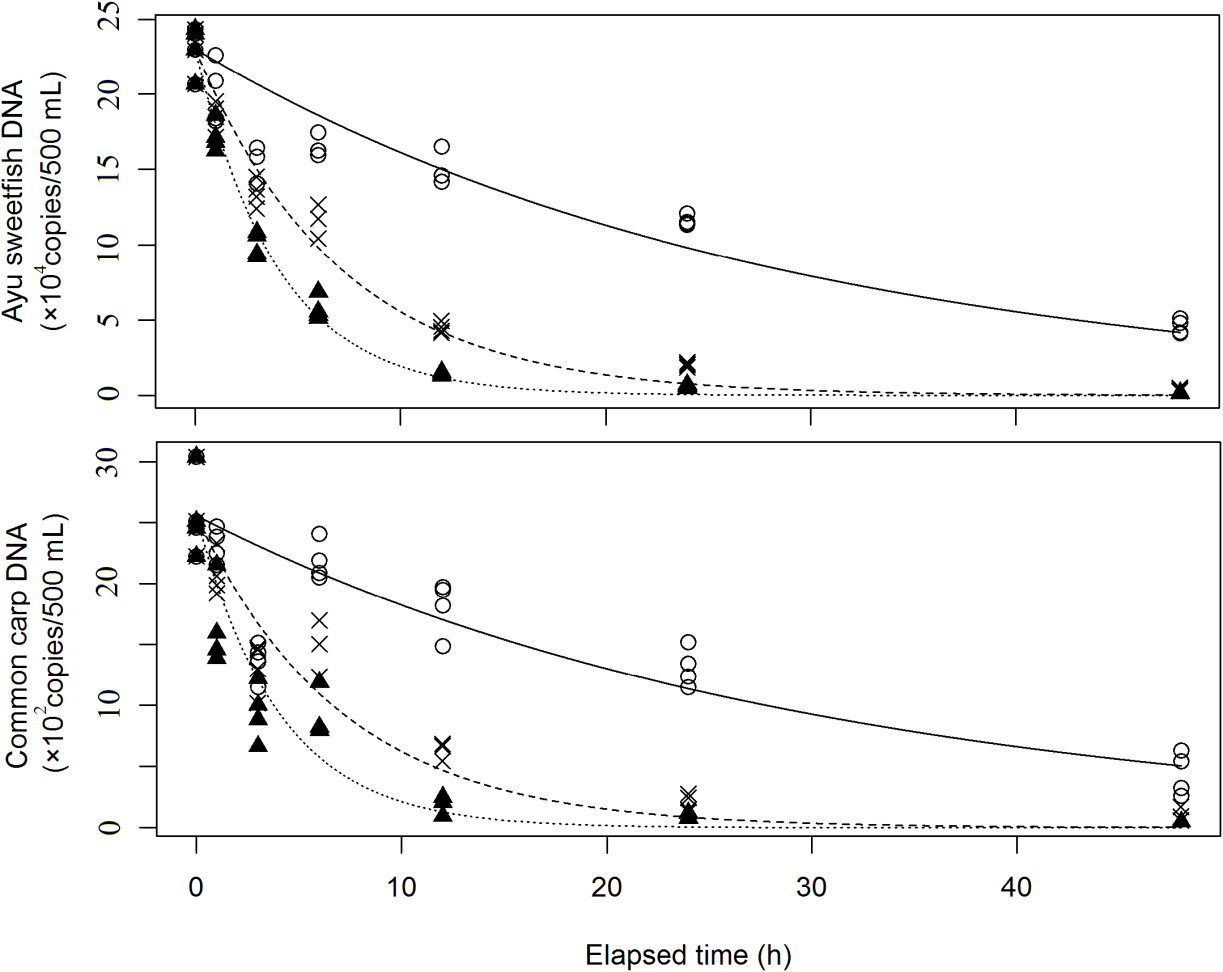
Time-dependent changes in eDNA concentration for ayu sweetfish and common carp. Circles, crosses, and triangles represent DNA concentrations for each species at 10°C, 20°C, and 30°C, respectively. Solid, dashed, and dotted lines represent nonlinear regression for 10°C, 20°C, and 30°C treatments, respectively.

In Experiment 1, the eDNA concentrations in water decreased exponentially with elapsed time. The initial eDNA concentrations of ayu sweetfish and carp in the initial sample water were 229,901 ± 16,763 and 2,558 ± 345 copies/500 mL (mean ± standard deviation), respectively. Environmental DNA degraded faster at a higher water temperature in both fish species (Fig 1, S1 Table). The coefficients of the interaction term of water temperature and time were different with all combinations of the three temperature controls for both ayu sweetfish and common carp (*p* < 0.01), except for temperatures between 20°C and 30°C in common carp (*p* = 0.07); there were stronger negative values at higher water temperatures (Table 1). The resulting nonlinear model fitted for ayu sweetfish and common carp is shown in Fig 1 (the equations of the full models are shown in Table 2). DNA degraded faster at a higher water temperatures for both species, so the half-decay times were shorter at higher water temperatures (Table 2). The constants *a* and *b* were estimated as *a* = −0.07081 and *b* = 0.01062 for ayu sweetfish and *a* = −0.07372 and *b* = 0.01075 for common carp.

**Table 1:** Results of generalized linear mixed-effects model analysis in Experiment 1 showing the effects of time and water temperature on eDNA concentration.

response variable	explanatory variable	coefficient	standard error
log (ayu sweetfish eDNA)			
	elapsed time (h)	–0.024	0.004
	elapsed time (h): water temperature 10°C	0 ^*a*^	
	elapsed time (h): water temperature 20°C	–0.058 ^*b*^	0.005
	elapsed time (h): water temperature 30°C	–0.091 ^*c*^	0.005
	Intercept	12.024	0.06
log (common carp eDNA)			
	elapsed time (h)	–0.027	0.005
	elapsed time (h): water temperature 10°C	0 ^*a*^	
	elapsed time (h): water temperature 20°C	–0.045 ^*b*^	0.006
	elapsed time (h): water temperature 30°C	–0.079 ^*b*^	0.008
	Intercept	7.533	0.07

All explanatory variables were significant at *p* = 0.05. The differences among coefficients of the interaction terms of elapsed time and water temperature were compared using the 95% confidence interval for the coefficients. The same superscript letters associated with the coefficients indicate statistical equivalence at *p* = 0.05 in each species. Note that the difference between 20°C and 30°C was marginally significant for common carp (*p* = 0.07).

**Table 2:** Full models and half-decay times for time-dependent degradation of eDNA as a result of nonlinear model fitting in Experiment 1.

species	full model	water temperature (°C)	half-decay time (h)
Ayu sweetfish	$N_{t}=229,901.2\times exp[-(0.01062\times k-0.07081)\times t]$		
		10	19.55
		20	4.89
		30	2.80
Common carp	$N_{t}=2,558.0\times exp[-(0.01075\times k-0.07372)\times t]$		
		10	20.50
		20	4.90
		30	2.80

**Fig 2:**
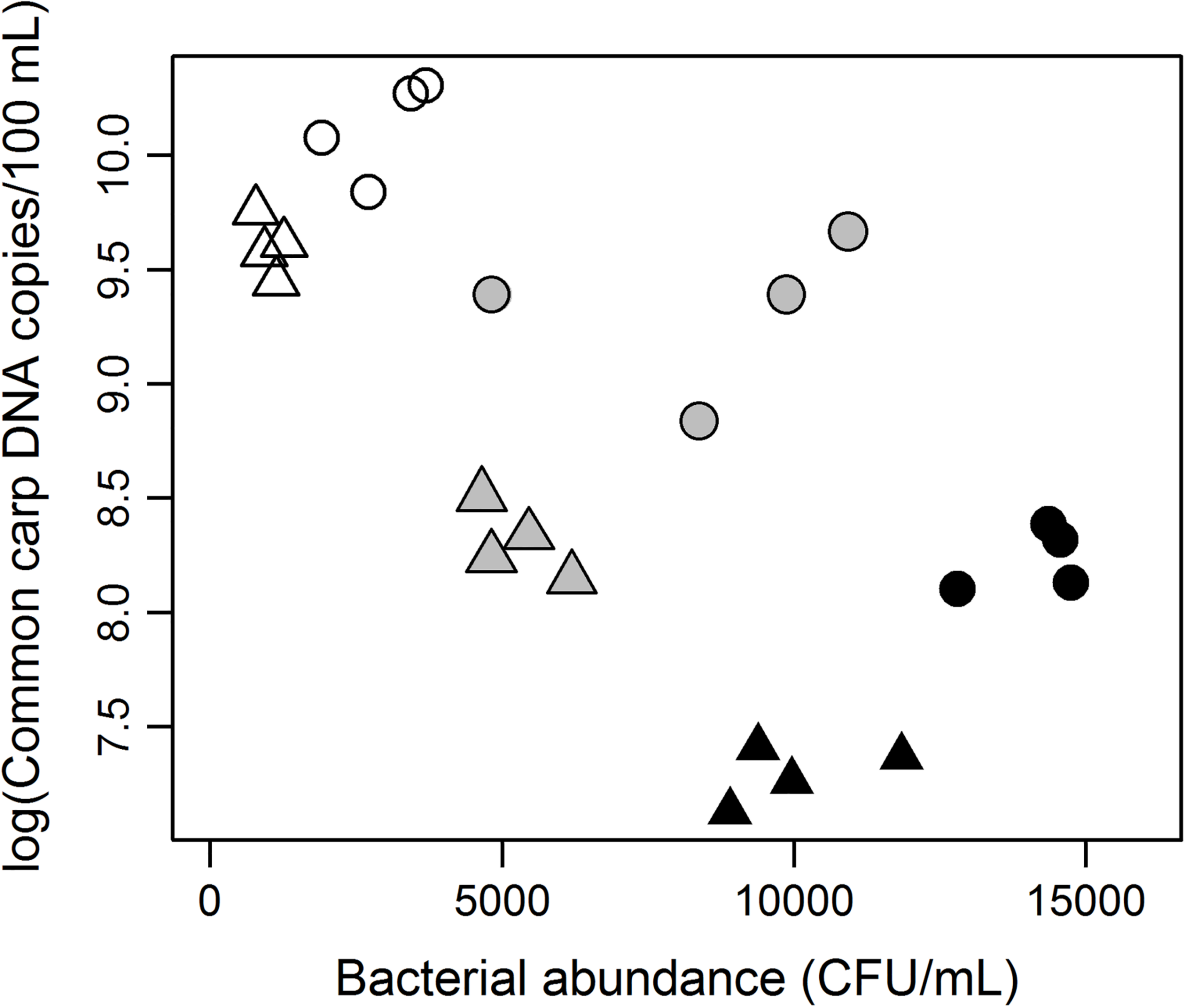
Relationship between bacterial abundance and DNA concentration of common carp in water after 12 and 24 h of incubation. Data for 12- and 24-h incubation trials are represented as circles and triangles, respectively. White, gray, and black plots represent water temperature settings of 10°C, 20°C, and 30°C, respectively.

In Experiment 2, we evaluated the relationship between the eDNA concentrations of common carp and bacterial abundances in bottles after 12 and 24 h of incubation. (Fig 2, S2 Table). The initial sample contained 647,803 ± 88,106 (mean ± standard deviation) copies/100 mL of common carp eDNA and 1,473 ± 188 (mean ± standard deviation) CFU/mL of bacteria (S2 Table). GLM analyses revealed that elapsed time and water temperature had significant negative effects on the degradation rate of common carp DNA (−5.35 × 10^−2^ and −1.35 × 10^−1^, respectively; *p* < 0.001 for both factors), but there was no significant effect on the carp DNA from bacterial abundance (5.99 × 10^−5^; *p* = 0.097). Elapsed time and water temperature also affected bacterial abundance (−2.57 × 10^2^ and 5.05 × 10^2^, respectively; *p* < 0.001 for both factors).

Ayu sweetfish and common carp DNAs were not detected in any equipment blanks or PCR negative controls in either experiment, indicating no cross-contamination during sample processing.

## Discussion

The present study showed that eDNA of ayu sweetfish and common carp in sample water degraded rapidly with time and that the degradation rate was affected by water temperature. Environmental DNA degraded faster at higher water temperatures, which is consistent with previous studies [17, 20]. The strong effect of water temperature on degradation rates suggests the importance of controlled storage temperatures during transportation; keeping water samples cool during transportation would retard the degradation of eDNA. To the best of our knowledge, an eDNA degradation model that explicitly incorporates water temperature has not been previously reported. To detect any differences among sites for sampling time or water temperature, it would be better to estimate initial eDNA concentrations in sample water at the time of collection. At this point, concentrations can be used for comparing biomass among sampling sites, which requires highly accurate estimations. The temperature-dependency of the eDNA degradation should also be considered when interpreting eDNA concentrations in the field to estimate target species biomass in different seasons or different times of day.

Water temperature-dependent degradation of eDNA in Experiment 1 led us to speculate that the degradation of eDNA was strongly influenced by the activity of bacterially-secreted DNA-degrading enzymes. However, in Experiment 2, we found that bacterial abundance did not have a significant effect on the degradation of eDNA, whereas elapsed time and water temperature did have significant effects on the eDNA degradation rates (Fig 2). In this study, we used one of the simplest traditional methods, i.e., standard culturing method, to estimate bacterial abundance, although this estimate does not include bacteria that are not amenable to culture. Other alternative methods such as flow cytometry or qPCR would provide a more comprehensive estimate for bacterial abundance; however, the culture-based method can provide a reliable index of bacterial abundance if the ratio of culturable bacteria to total bacteria has not changed among samples. Bacterial abundance and water temperature were highly correlated, i.e., bacterial abundance was higher at higher water temperatures. Ideally, the bacterial abundance and temperature should be independently controlled to explicitly determine their effects on the eDNA degradation; however, controlling bacterial abundances in field samples is complicated and multi-faceted. If we controlled the amount of bacteria in each sample by injecting a known amount of bacteria, we could separate the effects of bacterial abundance and water temperature on the degradation rate. Furthermore, a condition-dependent relationship is likely to exist between bacterial abundance and eDNA concentration; a positive correlation between bacterial abundance and eDNA concentration appeared if we focused on a single temperature treatment (for example 30°C), whereas a negative correlation appeared when three temperature treatments were taken into account altogether (Fig 2). Many complex processes would be involved in eDNA degradation such as the dynamics of bacterial populations, the production of DNA-degrading enzymes by bacteria, and the enzymatic degradation rates of eDNA; therefore, bacterial populations can influence eDNA concentrations directly or indirectly. These complex mechanisms would be a source of the condition-dependent relationship between eDNA concentration and bacterial abundance. Additionally, eDNA in field water exists in various states such as free-floating DNA and DNA contained in organelles and cells [6,23]. If most of the eDNA is contained in organelles and cells, as suggested by Turner et al. [23] and Tsuji et al. [6], it is plausible that the bacterial effects would be slower and the rate of eDNA degradation would be slower. It would be ideal to use eDNA samples reflecting the realistic components of eDNA in natural samples; however, we used purified DNA instead in Experiment 2 because it was difficult to isolate eDNA components from field water without bacterial contamination. Further studies on the effects of bacteria on eDNA degradation would be desirable, paying attention to the state of the eDNA and the condition-dependent effect of bacteria.

Eichmiller et al. [17] reported the degradation constant *k*, equivalent to *β* (/h) in Thomsen et al. [15], for common carp eDNA at four different water temperatures using a simple degradation model reported by Thomsen et al. [15]. The constants were 0.015 ± 0.00083, 0.078 ± 0.0046, 0.10 ± 0.0063, and 0.10 ± 0.0063 (average ± standard error, /h) at 5°C, 15°C, 25°C, and 35°C, respectively. In the degradation model constructed in this study, the degradation rate was denoted as *bk + a* (/h), which includes the effect of water temperature and is equivalent to *k* in Eichmiller et al. [17] when the water temperature factor (*k*) is substituted. As a result, the degradation rate at each temperature can be calculated as −0.020, 0.088, 0.20, and 0.30/h at 5°C, 15°C, 25°C, and 35°C, respectively. Compared with the rates of Eichmiller et al. [17], the estimated degradation rates in this study were higher, at 15°C, 25°C, and 35°C (especially at 35°C, with a rate three times that in the previous study). Suspended humic materials and clays can slow down DNA degradation due to their binding effect on DNA fragments, resulting in protection from enzymatic degradation [24]. In addition, the lower and higher degradation rates at lower and higher water temperature conditions, respectively, seem to be mainly due to the adoption of the linear function to approximate the effect of water temperature. It might be unreasonable to incorporate the linear function to approximate the water temperature effect; however, the present model would not lose its practicability since the extremely warm condition in which the degradation constant was overestimated is not commonly observed in the temperate zone. For example, the temperature range of the surface water in the Yasu River, Shiga, Japan, is 4°C to 28°C [25].

Knowledge of the effects of water qualities (temperature, pH, conductivity) on eDNA degradation compared to those in field water, such as suspended humic materials, suspended solids, and microbial abundance is required for better estimates of macro species distribution and biomass based on eDNA concentrations [9]. The present study showed that the eDNA degradation rate is strongly influenced by water temperature and we cannot ignore this effect in efforts to improve the accuracy of quantification of eDNA. Because the degradation of eDNA is rapid and sensitive to environmental factors, further clarification of the relationship between eDNA degradation and other environmental factors will increase the reliability of biomass estimations based on eDNA analysis.

## Supporting information

S1 Table All raw data obtained in Experiment 1. The eDNA concentrations of ayu sweetfish and common carp at each water temperature and elapsed time.
(XLSX)

S2 Table All raw data obtained in Experiment 2. The eDNA concentrations of common carp and bacterial abundance at each water temperature and elapsed time.
(XLSX)

## References

[1] Ficetola GF, Miaud C, Pompanon F, Taberlet P. Species detection using environmental DNA from water samples. Biol Lett. 2008;4: 423–425. doi: 10.1098/rsbl.2008.0118 18400683 10.1098/rsbl.2008.0118 PMC2610135

[2] Jerde CL, Mahon AR, Chadderton WL, Lodge DM. “Sight-unseen” detection of rare aquatic species using environmental DNA. Conserv Lett. 2011;4: 150–157.

[3] Lodge DM, Turner CR, Jerde CL, Barnes MA, Chadderton L, Egan SP, et al Conservation in a cup of water: estimating biodiversity and population abundance from environmental DNA. Mol Ecol. 2012;21: 2555–2558. doi: 10.1111/j.1365-294X.2012.05600.x 22624944 10.1111/j.1365-294X.2012.05600.x PMC3412215

[4] Minamoto T, Yamanaka H, Takahara T, Honjo MN, Kawabata Z. Surveillance of fish species composition using environmental DNA. Limnol. 2012;13: 193–197.

[5] Kelly RP, Port JA, Yamahara KM, Crowder LB. Using environmental DNA to census marine fishes in a large mesocosm. PLOS ONE. 2014;9: e86175 doi: 10.1371/journal.pone.0086175 24454960 10.1371/journal.pone.0086175 PMC3893283

[6] Tsuji S, Yamanaka H, Minamoto T. Effects of water pH and proteinase K treatment on the yield of environmental DNA from water samples. Limnol. 2016.

[7] Takahara T, Minamoto T, Doi H. Using environmental DNA to estimate the distribution of an invasive fish species in ponds. PLOS ONE. 2013;8: e56584 doi: 10.1371/journal.pone.0056584 23437177 10.1371/journal.pone.0056584 PMC3577852

[8] Ushio M, Fukuda H, Inoue T, Makoto K, Kishida O, Sato K, et al. Environmental DNA enables detection of terrestrial mammals from forest pond water. bioRxiv. 2016. 10.1111/1755-0998.12690 28603873

[9] Yamamoto S, Minami K, Fukaya K, Takahashi K, Sawada H, Murakami H, et al Environmental DNA as a ‘Snapshot’ of Fish Distribution: A case study of Japanese Jack Mackerel in Maizuru Bay, Sea of Japan. PLOS ONE. 2016;11(3): e0149786 doi: 10.1371/journal.pone.0149786 26933889 10.1371/journal.pone.0149786 PMC4775019

[10] Yamanaka H, Minamoto T. The use of environmental DNA of fishes as an efficient method of determining habitat connectivity. Ecol Indic. 2016;62: 147–153.

[11] Takahara T, Minamoto T, Yamanaka H, Doi H, Kawabata Z. Estimation of fish biomass using environmental DNA. PLOS ONE. 2012;7(4): e35868 doi: 10.1371/journal.pone.0035868 22563411 10.1371/journal.pone.0035868 PMC3338542

[12] Doi H, Inui R, Akamatsu Y, Kanno K, Yamanaka H, Takahara T, et al Environmental DNA analysis for estimating the abundance and biomass of stream fish. Freshwater Biol. 2016.

[13] Sigsgaard EE, Carl H, Møller PR, Thomsen PF. Monitoring the near-extinct European weather loach in Denmark based on environmental DNA from water samples. Biol Conserv. 2015;183:46–52.

[14] Fukumoto S, Ushimaru A, Minamoto T. A basin-scale application of environmental DNA assessment for rare endemic species and closely related exotic species in rivers: a case study of giant salamanders in Japan. J Appl Ecol. 2015;52: 358–365.

[15] Thomsen PF, Kielgast J, Iversen LL, Møller PR, Rasmussen M, Willerslev E. Detection of a Diverse Marine Fish Fauna Using Environmental DNA from Seawater Samples. PLOS ONE 2012; 7(8): e41732 doi: 10.1371/journal.pone.0041732 22952584 10.1371/journal.pone.0041732 PMC3430657

[16] Maruyama A, Nakamura K, Yamanaka H, Kondoh M, Minamoto T. The Release Rate of Environmental DNA from Juvenile and Adult Fish. PLOS ONE. 2014;9: e114639 doi: 10.1371/journal.pone.0114639 25479160 10.1371/journal.pone.0114639 PMC4257714

[17] Eichmiller J, Best SE, Sorensen PW. Effects of temperature and trophic state on degradation of environmental DNA in lake water. Environ Sci Technol. 2016;50(4): 1859–1867. doi: 10.1021/acs.est.5b05672 26771292 10.1021/acs.est.5b05672

[18] Barnes MA, Turner CT, Jerde CL, Renshaw MA, Chadderton WL, Lodge DM. Environmental Conditions Influence eDNA Persistence in Aquatic Systems. Environ Sci Technol.2014;48:1819–1827. doi: 10.1021/es404734p 24422450 10.1021/es404734p

[19] Pilliod DS, Goldberg CS, Arkle RS, Waits LP. Factors influencing detection of eDNA from a stream-dwelling amphibian. Mol Ecol Resour. 2014;14: 109–116. doi: 10.1111/1755-0998.12159 24034561 10.1111/1755-0998.12159

[20] Strickler KM, Fremier AK, Goldberg CS. Quantifying effects of UV-B, temperature, and pH on eDNA degradation in aquatic microcosms. Biol Conserv. 2015;183: 85–92.

[21] R Core Team. R: A language and environment for statistical computing; 2014. Available from: http://www.r-project.org/.

[22] Hothorn T, Bretz F, Westfall P. Simultaneous Inference in General Parametric Models. Biometrical J. 2008;50(3): 346–363. 10.1002/bimj.200810425 18481363

[23] Turner CR, Barnes MA, Xu CCY, Jones SE, Jerde CL, Lodge DM. Particle size distribution and optimal capture of aqueous macrobial eDNA. Methods Ecol Evol 2014;5: 676–684.

[24] Stotzky G. Persistence and Biological Activity in Soil of Insecticidal Proteins from Bacillus thuringiensis and of Bacterial DNA Bound on Clays and Humic Acids. J Env Qual. 2000;29: 691–705.

[25] Endoh S, Okumura Y, Kawashima M, Fukuyama N, Onishi Y, Makamura N, et al Dispersion of Yasu River Water in Lake Biwa. Jpn. J Limnol. 2007;68:15–27.

